# Biological and Clinical Significance of *MAD2L1* and *BUB1*, Genes Frequently Appearing in Expression Signatures for Breast Cancer Prognosis

**DOI:** 10.1371/journal.pone.0136246

**Published:** 2015-08-19

**Authors:** Zhanwei Wang, Dionyssios Katsaros, Yi Shen, Yuanyuan Fu, Emilie Marion Canuto, Chiara Benedetto, Lingeng Lu, Wen-Ming Chu, Harvey A. Risch, Herbert Yu

**Affiliations:** 1 Cancer Epidemiology Program, University of Hawaii Cancer Center, Honolulu, Hawaii, United States of America; 2 Department of Surgical Sciences, Gynecologic Oncology, Azienda Ospedaliero-Universitaria Città della Salute, Turin, Italy; 3 Department of Chronic Disease Epidemiology, Yale School of Public Health, New Haven, Connecticut, United States of America; 4 Cancer Biology Program, University of Hawaii Cancer Center, Honolulu, Hawaii, United States of America; University of North Carolina School of Medicine, UNITED STATES

## Abstract

To investigate the biologic relevance and clinical implication of genes involved in multiple gene expression signatures for breast cancer prognosis, we identified 16 published gene expression signatures, and selected two genes, *MAD2L1* and *BUB1*. These genes appeared in 5 signatures and were involved in cell-cycle regulation. We analyzed the expression of these genes in relation to tumor features and disease outcomes. *In vitro* experiments were also performed in two breast cancer cell lines, MDA-MB-231 and MDA-MB-468, to assess cell proliferation, migration and invasion after knocking down the expression of these genes. High expression of these genes was found to be associated with aggressive tumors and poor disease-free survival of 203 breast cancer patients in our study, and the association with survival was confirmed in an online database consisting of 914 patients. *In vitro* experiments demonstrated that lowering the expression of these genes by siRNAs reduced tumor cell growth and inhibited cell migration and invasion. Our investigation suggests that MAD2L1 and BUB1 may play important roles in breast cancer progression, and measuring the expression of these genes may assist the prediction of breast cancer prognosis.

## Introduction

Accurately predicting the prognosis of breast cancer remains a significant challenge [1]. Many clinical, pathologic and molecular markers have been identified for breast cancer prognosis, including disease stage, tumor grade and histology, lymph node involvement, hormone/growth factor receptor status and recently molecular subtypes, but none of them provides ideal accuracy in predicting prognosis and treatment response. High-throughput analyses, based on microarray chips and other technologies, are used to determine gene expression “signatures” for improved accuracy of prognosis. The first such signature was reported in 2002, which was on based on the expression of 70 genes using a DNA-microarray technology [2]. Since then, more than a dozen of gene expression signatures have been published for breast cancer prognosis [3–15]. Interestingly, when comparing the genes in the signatures, only a small number are shared by these signatures, and most of the genes are not overlapping. Reasons for this disparity in gene lists are probably multiple, including diverse patient populations, disease characteristics, analytical technologies, tissue preservation and preparation methods, and some degree of false positivity. Because of the inconsistency of gene inclusion across signatures as well as the feasibility and cost of using such signatures in patient management, many of the expression signatures have not been confirmed in prospective studies. To date, only two prognostic signatures [2, 16] are being evaluated in clinical trials for utility [17, 18], but the results will not be available until 2020.

While many studies are still aiming to develop new methods and techniques to improve the accuracy of prediction with the use of gene signatures, the biological relevance or pathologic involvement of the genes in signatures are often overlooked. Usually, the genes in a signature involve a number of biological processes and functions, making it difficult to identify key components that may drive tumor progression. For example, the first 70-gene signature [2] contains genes involved in multiple biological activities, including cell cycle regulation, cell invasion and metastasis, angiogenesis, and signal transduction. Which process play key roles in tumor progression is unclear. Furthermore, the effects on tumor cells of many genes in the signatures are unknown. Thus, in an attempt to address some of these issues, we identified and compared genes from 16 publications that have reported gene-expression signatures for breast cancer prognosis. From the genes that appeared in multiple expression signatures, we selected two, *MAD2L1* (Mitotic Arrest Deficient 2-like 1) and *BUB1* (Budding Uninhibited by Benzimidazoles 1), for further validation of their associations with patient survival in our own and other studies, as well as for *in vitro* evaluation of their biological involvements in breast cancer.

## Material and Methods

### Selection of gene expression signatures

We searched the PubMed database (US National Library of Medicine) using the phrases “breast cancer”, “gene expression”, “signature”, and “survival”. The search was updated in May 2015. The studies selected for our analysis were based on the following conditions: a) it was an initial report of a gene signature associated with breast cancer survival; b) an entire list of genes involved in the signature was reported; and c) gene expression data were generated from microarray analysis. Following the criteria, a total of 14 studies (or signatures) were selected [2–15, 18, 19]. Two additional studies by van Vliet et al. [20] and Dai et al. [21] were also included in our analysis. These studies analyzed the same data from Van’s Veer [2], but used very different strategies and generated different signatures with regard to the lists of genes. Therefore, they were considered as independent studies. The study conducted by Van’s Veer et al [2] identified a 231-gene signature in the initial stage. We used the genes in the initial signature for our analysis. A well-known EndoPredict signature based on RT-qPCR analysis was also included in our study [12].

### Breast cancer patients

We recruited 203 female patients diagnosed with primary breast cancer from the University Hospital at University of Turin between January 1998 and July 1999. The patients were enrolled in the study before surgery and were followed through February 2007. The study was approved by the ethical review committee of University Hospital at University of Turin. All participants provided written informed consent. Fresh breast tumor samples removed during surgery were snap-frozen in liquid nitrogen immediately after resection and stored at -80°C until analysis. Clinical and pathological characteristics of the patients are shown in the result section.

### RNA extraction and analysis

Protocols for RNA extraction and gene expression analysis have been described elsewhere [22]. Briefly, total RNA was extracted from fresh-frozen tumor samples according to the conventional phenol-chloroform method following manual pulverization of tissue specimens in liquid nitrogen. The RNA samples were purified and concentrated by RNeasy MinElute Cleanup Kit (Qiagen Inc., Valencia, CA). Concentrations and integrities of total RNA were analyzed using the RNA 6000 Nano LabChip Kit and Agilent 2100 Bioanalyzer. Microarray analyses were performed using the Illumina Expression BeadChip (HumanRef-8 v3) following the manufacturer’s protocol.

### Antibodies

Antibodies used for western blot analysis were purchased from various companies. Anti-MAD2L1 (D8A7, #4636) antibody and horseradish peroxidase (HRP)-conjugated secondary antibody were purchased from Cell Signaling Technology (Danvers, MA), anti-BUB1 antibody (ab54893) was from Abcam (Cambridge, MA), and anti-β-actin antibody (A2228) was from Sigma-Aldrich (St. Louis, MO).

### Cell lines and cultures

Human breast cancer cell lines MDA-MB-231 (MB231) and MDA-MB-468 (MB468) used for our *in vitro* experiments were supplied by Dr. Richard Yip at University of Hawaii Cancer Center, who obtained them from NCI as part of the NCI-60 DTP Human Tumor Cell Screening Panel. The cells were cultured in Dulbecco's Modified Eagle Medium (DMEM) supplemented with 10% fetal bovine serum (FBS) and 100 units/ml of penicillin/streptomycin (Pen/Strep) at 37°C in a humidified atmosphere of 5% CO_2_.

### Western blots

In siRNA knockdown experiments, protein expression was analyzed by western blot. Cells were treated with a lysis buffer [1% Triton x-100, 150 mM NaCl, 50 mM Tris-Cl, pH 7.4, 0.5 mM EDTA, 1 mM sodium orthovanadate, 5 mM beta glycerol phosphate, and 1x protease inhibitor cocktail (Roche, Indianapolis, IN)] after transfection with indicated siRNAs for 36 h, and protein concentration was measured. Proteins (40–60 μg) from the cell lysates were separated by SDS-PAGE (10% sodium dodecyl sulfate-polyacrylamide gel electrophoresis) under denaturing conditions, and then transferred to polyvinylidene difluoride (PVDF) membranes (Millipore, Billerica, MA). After transferring, the membranes were blocked with 5% non-fat milk for 45 minutes, and then incubated with a primary antibody followed by incubation with a secondary antibody. The signals were detected by the enhanced chemiluminescence system (ECL) as described by the manufacturer (Pierce, Rockford, IL).

### siRNA transfection

The ON-TARGET plus SMARTpool siRNA of human *MAD2L1* and *BUB1* and negative control ON-TARGET plus Non-targeting Pool were purchased from Dharmacon (Lafayette, CO). The sequences of *MAD2L1* and *BUB1* siRNAs as well as control siRNAs are (a) ON-TARGET plus SMARTpool Human *MAD2L1* siRNA: UUACUCGAGGCAGAAAUA (siMAD2L1-1), CUACUGAUCUUGAGCUCAU (siMAD2L1-2), GGUUGUAGUUAUCUCAAAU (siMAD2L1-3) and GAAAUCCGUUCAGUGAUCA (siMAD2L1-4); (b) ON-TARGET plus SMARTpool Human *BUB1* siRNA: CGAAGAGUGAUCACGAUUU (siBUB1-1), CAAAGAAGGGUGUGAAACA (siBUB1-2), GAAUGUAAGCGUUCACGAA (siBUB1-3), and GCAACAAACCAUGGAACUA (siBUB1-4); and (c) ON-TARGET plus Non-targeting siRNA: UGGUUUACAUGUCGACUAA (siRNA-1), UGGUUUACAUGUUGUGUGA (siRNA-2), UGGUUUACAUGUUUUCUGA (siRNA-3), and UGGUUUACAUGUUUUCCAU (siRNA-4). Cells were seeded onto 6-well plates in antibiotic-free medium plus 10% FBS overnight. When 70% confluent, cells were transfected with Lipofectamine RNAiMAX (Invitrogen) according to the manufacturer’s protocol. The final concentration of siRNAs was 50 nM. After transfection, cells were cultured for 36 hours, and then analyzed by western blot for protein expression of MAD2L1 and BUB1, as well as cell proliferation, *in vitro* migration and invasion assays.

### Cell proliferation assay

Cells were plated onto 96-well plates at a density of 3 x 10^3^ cells per well, and cultured in the complete medium at 37°C under a humidified atmosphere containing 5% CO_2_. At 0, 24, 48, and 72 hours of incubation, cell proliferation reagent WST-1 was added into the wells in accordance with the manufacturer’s instructions (Roche, Penzberg, Germany). Cells were continuously incubated for 2 hours with WST-1, and then the color in each well of the plate was measured by a microplate spectrophotometer (Biotek Synergy 2, Winooski, VT) at the 450 nm wavelength. The light absorbance was proportional to the numbers of cells in each well. The results of measurement at different time points of incubation were referenced to the measurement at 0 hours of incubation. Each proliferation assay was performed in triplicate.

### Transwell migration and invasion assays

*In vitro* cell migration and invasion assays were performed in 24-well plates with the Costar Transwell permeable membrane support with 8.0-μm pore size (Corning, NY). In the cell invasion assay, 200 μl control and siRNA knockdown cells in serum-free medium (1 x 10^4^ cells per well) were seeded in the upper chambers coated with growth-factor-reduced Matrigel, 1 mg/ml (BD Pharmingen, San Diego, CA). The lower chambers were filled with 600 μl culture medium containing 10% FBS. Cell migration assays were performed similarly but without Matrigel coating. Cells invading or migrating to the underside of the filter membrane were stained with HEME 3 Solution (Fisher Diagnostics, Middletown, VA) after 36 hours of culture, and then counted using an Olympus CKX41 microscope with an Infinity 2 camera. All experiments were repeated three times with triplicate wells.

### Statistical analysis

DAVID Bioinformatics Resources v6.7 (http://david.abcc.ncifcrf.gov) was used for the pathway enrichment analysis, with recommended standard parameters selected in the analysis [23, 24]. KEGG (Kyoto Encyclopedia of Genes and Genomes) database (http://www.genome.ad.jp/kegg/) was employed for the analysis of protein functions in different pathways. All the microarray data were pre-processed by the BeadStudio Software and analyzed using the R statistical software and Bioconductor [25]. The Lumi R package was used to normalize the data [26]. Wilcoxon rank-sum test was performed to assess the association of gene expression and clinicopathologic characteristics of breast cancer patients. Kaplan-Meier survival analysis and Cox proportional hazards regression models were used to analyze the association of gene expression with breast cancer survival. Log-rank test was performed for Kaplan-Meier survival curve comparisons. Hazards ratios (HRs) and 95% confidence intervals (CIs) were calculated in the Cox regression analysis, and both univariate and multivariate models were developed. In multivariate analysis, HRs were adjusted for patient age at surgery, tumor grade, disease stage, and estrogen receptor (ER) and progesterone receptor (PR) status. Two types of survival outcomes were considered in survival analysis, overall survival (OS) defined as the time between the date of surgery and date of death or last follow-up, and relapse-free survival (RFS) defined as the period from surgery to recurrence or last follow-up. *MAD2L1* and *BUB1* expression were categorized into low, medium and high based on their tertile distributions. To validate the associations of *MAD2L1* and *BUB1* expression with breast cancer survival, an online tool named Gene expression-based Outcome for Breast cancer Online (GOBO) (http://co.bmc.lu.se/gobo) was used [27]. All of the values presented in the figures of *in vitro* experiments are means and standard deviations. Two-tailed Student *t* test was performed to determine the differences in means between groups, and *P*<0.05 was considered statistically significant. All statistical analyses were performed using the Statistical Analysis System, version 9.2, (SAS Institute Inc., Cary, NC) and R software (version 3.0.2).

## Results

### Genes in 16 reported expression signatures

Studies reported gene expression signatures in association with breast cancer prognosis are listed in S1 Table. These signatures included a total of 1,399 distinct genes. Of them, 1,138 were found to have official names. One gene, *BIRC5* (also known as Survivin), was included in 7 signature lists, another, *MYBL2*, was in 6 lists, 5 genes (*BUB1*, *CENPF*, *MAD2L1*, *RRPM2*, *PRC1*) were in 5 lists, and 12 genes were in 4 lists. The complete list of genes appearing in 3 or more signature lists is provided in S2 Table. Using the DAVID Bioinformatics Resources v6.7 to analyze the 1,138 genes for pathway enrichment, we found four significantly enriched pathways. Cell cycle was the most significant one (Benjamini-Hochberg corrected p = 10^−9.5^), followed by p53 signaling pathway (p = 10^−4.2^), pathways in cancer (p = 10^−3.6^), and DNA replication (p = 0.016). We found that three genes involved in multiple signatures were also associated with survival outcomes in our study, including MAD2 mitotic arrest deficient-like 1 (*MAD2L1*), pituitary tumor-transforming gene-1 (*PTTG1*), and budding uninhibited by benzimidazole 1 (*BUB1*). All of these genes were involved in the cell cycle pathway. Since the effects of *PTTG1* on breast cancer cells have been studied previously by Yoon at el. [28], we focused our analyses on *MAD2L1* and *BUB1*.

### Associations of *MAD2L1* and *BUB1* expression with clinical and pathologic features

Using the gene expression data from our microarray analysis of breast cancer [22], we examined the associations of *MAD2L1* and *BUB1* expression with tumor features and disease outcomes. Table 1 shows the clinical and pathologic characteristics of breast cancer patients in association with *MAD2L1* and *BUB1* expression. Patients with PR negative, ER negative, and high grade tumors had higher expression of *MAD2L1* and *BUB1* compared to those with PR positive, ER positive, and low grade tumors, respectively. Patient age, disease stage and lymph node involvement were not associated with *MAD2L1* or *BUB1* expression.

**Table 1:** Associations of *MAD2L1* and *BUB1* expression with clinical and molecular characteristics of breast cancer.

Variables	*MAD2L1*				*BUB1*			
	Low No. (%)	Middle No. (%)	High No. (%)	*P*	Low No. (%)	Middle No. (%)	High No. (%)	*P*
**Age at surgery**				0.375				0.113
**≤57.11**	35(34.31)	30(29.42)	37(36.27)		37(36.27)	27(26.48	38(37.25)	
**>57.11**	33(32.35)	39(38.24)	30(29.41)		31(30.39)	41(40.20)	30(29.41)	
**Estrogen receptor**				0.027				0.00036
Positive	47(37.01)	47(37.01)	33(25.98)		52(40.94)	45(35.43)	30(23.62)	
Negative	21(27.27)	22(28.57)	34(44.16)		16(20.78)	23(29.87)	38(49.35)	
**Progesterone receptor**				0.012				0.000002
Positive	42(41.18)	36(35.29)	24(23.53)		49(48.04)	34(33.33)	19(18.63)	
Negative	25(25.51)	32(32.65)	41(41.84)		18(18.37)	33(33.67)	47(47.96)	
**Lymph node status**				0.254				0.662
Positive	31(31.31)	39(39.39)	29(29.29)		30(30.30)	35(35.35)	34(34.34)	
Negative	37(35.24)	30(28.57)	38(36.19)		38(36.19)	33(31.43)	34(32.38)	
**Grade**				0.000001				< .000001
1	18(69.23)	4(15.38)	4(15.38)		15(57.69)	7(26.92)	4(15.38)	
2	29(37.18)	33(42.31)	16(20.51)		38(48.72)	25(32.05)	15(19.23)	
3	20(20.41)	31(31.63)	47(47.96)		13(13.27)	36(36.73)	49(50.00)	
**Disease stage**				0.237				0.093
I	28(42.42)	22(33.33)	16(24.24)		26(39.39)	26(39.39)	14(21.21)	
II	34(30.63)	36(32.43)	41(36.94)		36(32.43)	34(30.63)	41(36.94)	
III, IV	6(22.22)	11(40.74)	10(37.04)		6(22.22)	8(29.63)	13(48.15)	

To evaluate whether *MAD2L1* and *BUB1* are over-expressed in breast cancer, a dataset (GSE37751) was downloaded from the Gene Expression Omnibus (GEO) website (www.ncbi.nlm.nih.gov/geo). This dataset included gene expression data from 61 breast cancer and 47 adjacent non-tumor tissues analyzed by the GeneChip Human Gene 1.0 ST arrays (Affymetrix, Santa Clara, CA). The expression data were processed by the RMA algorithm using the Affymetrix Expression Console software [29]. Both *MAD2L1* and *BUB1* expression were found to be higher in tumor than in adjacent non-tumor tissues (5.97±0.66 versus 5.36±0.38 for *MAD2L1*, 6.80±1.14 versus 5.50±0.74 for *BUB1*, respectively, and both P<0.0001) in this database. Similar differences were also observed in another GEO dataset (GSE29044) [30].

### Associations of *MAD2L1* and *BUB1* expression with overall and relapse-free survival

**Fig 1:**
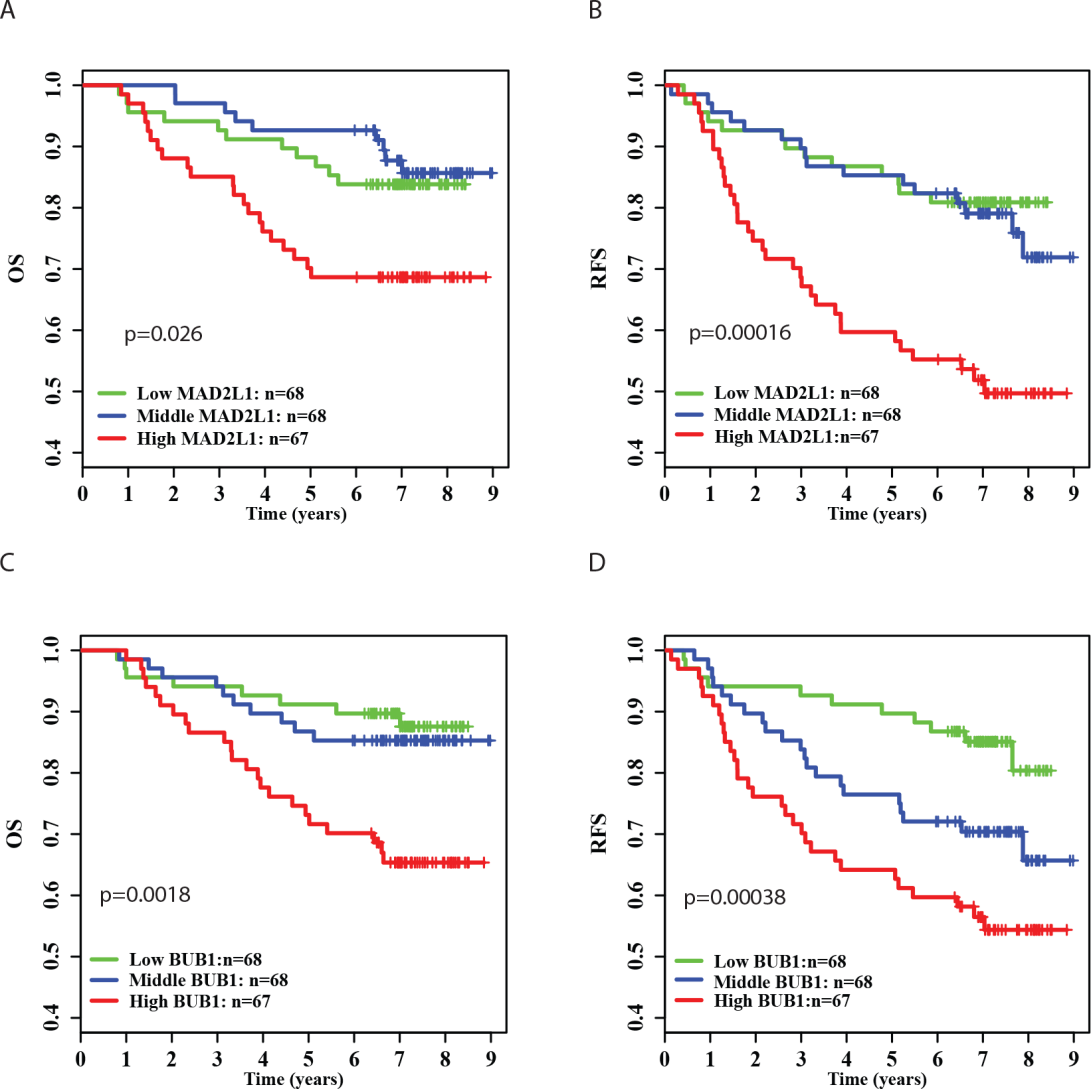
Kaplan-Meier survival curves by different levels of *MAD2L1* and *BUB1* expression in 203 breast cancer patients. A) Overall survival (OS) by low, medium and high *MAD2L1* expression; B) Relapse-free survival (RFS) by low, medium and high *MAD2L1* expression; C) Overall survival (OS) by low, medium and high *BUB1* expression; (D) Relapse-free survival (RFS) by low, medium and high *BUB1* expression.

**Fig 2:**
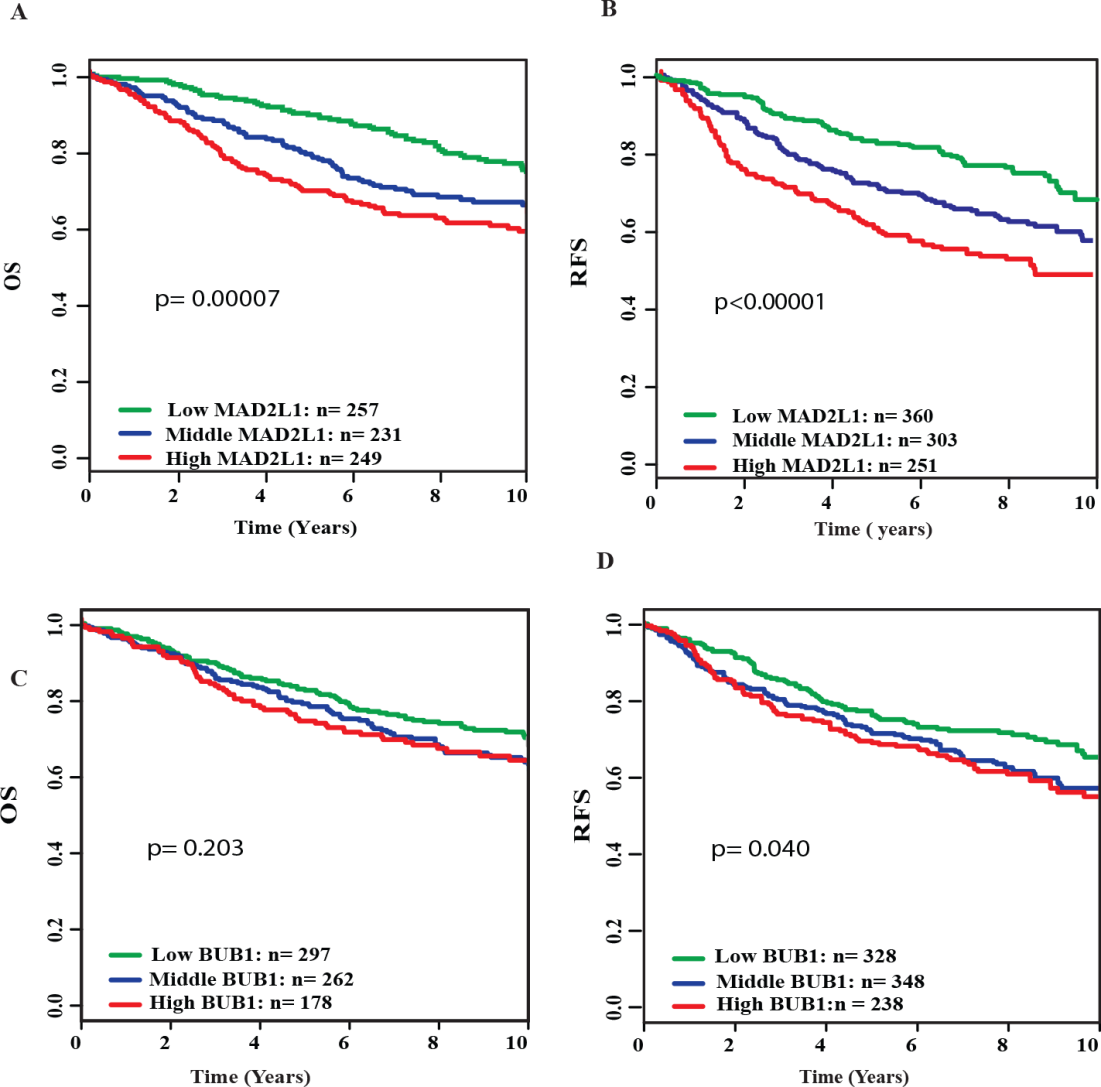
Kaplan-Meier survival curves by different levels of *MAD2L1* and *BUB1* expression in the GOBO database. A) Overall survival (OS) by low, medium and high *MAD2L1* expression; B) Relapse-free survival (RFS) by low, medium and high *MAD2L1* expression; C) Overall survival (OS) by low, medium and high *BUB1* expression; (D) Relapse-free survival (RFS) by low, medium and high *BUB1* expression.

We further investigated the associations of *MAD2L1* and *BUB1* expression with breast cancer survival. Fig 1 shows that high expression of *MAD2L1* was significantly associated with increased risk of disease recurrence and death. Patients with high tumor expression of *BUB1* also had poorer relapse-free and overall survival compared to those with low tumor expression. The associations of *MAD2L1* and *BUB1* expression with relapse-free survival remained statistically significant after adjusting for age at surgery, ER and PR status, tumor grade, and disease stage (Table 2). The relationships of breast cancer survival with *MAD2L1* and *BUB1* expression were also analyzed in the GOBO database consisting of 914 tumor samples from 11 publically available microarray datasets. The results of the GOBO database analysis were similar to the findings of our study. High expression of *MAD2L1* and *BUB1* were significantly associated with poor relapse-free survival (Fig 2B and 2D). High *MAD2L1* expression was also associated with poor overall survival (Fig 2A).

**Table 2:** Associations of *MAD2* and *BUB1* expression with overall and relapse-free survival.

Variables	Unadjusted						Adjusted*					
	Overall			Relapse-free			Overall			Relapse-free		
	HR	95% CI		HR	95% CI		HR	95% CI		HR	95% CI	
***MAD2L1***												
low	1			1			1			1		
Mid	0.786	0.326	1.896	1.209	0.581	2.514	0.572	0.231	1.417	0.877	0.41	1.876
High	**2.163**	**1.042**	**4.487**	**3.177**	**1.671**	**6.042**	1.528	0.673	3.472	**2.08**	**1.027**	**4.214**
P for trend		**0.0257**			**0.00016**			0.223			**0.0183**	
***BUB1***												
low	1			1			1			1		
Mid	1.269	0.501	3.216	2.067	0.996	4.289	1.214	0.439	3.355	1.652	0.744	3.67
High	**3.263**	**1.459**	**7.298**	**3.378**	**1.691**	**6.746**	2.543	0.96	6.734	**2.329**	**1.037**	**5.231**
P for trend		**0.0019**			**0.00038**			**0.033**			**0.0054**	

* Adjusted for age at surgery, tumor grade, disease stage, ER status, and PR status.

### Suppression of *MAD2L1* and *BUB1* expression in breast cancer cell lines

**Fig 3:**
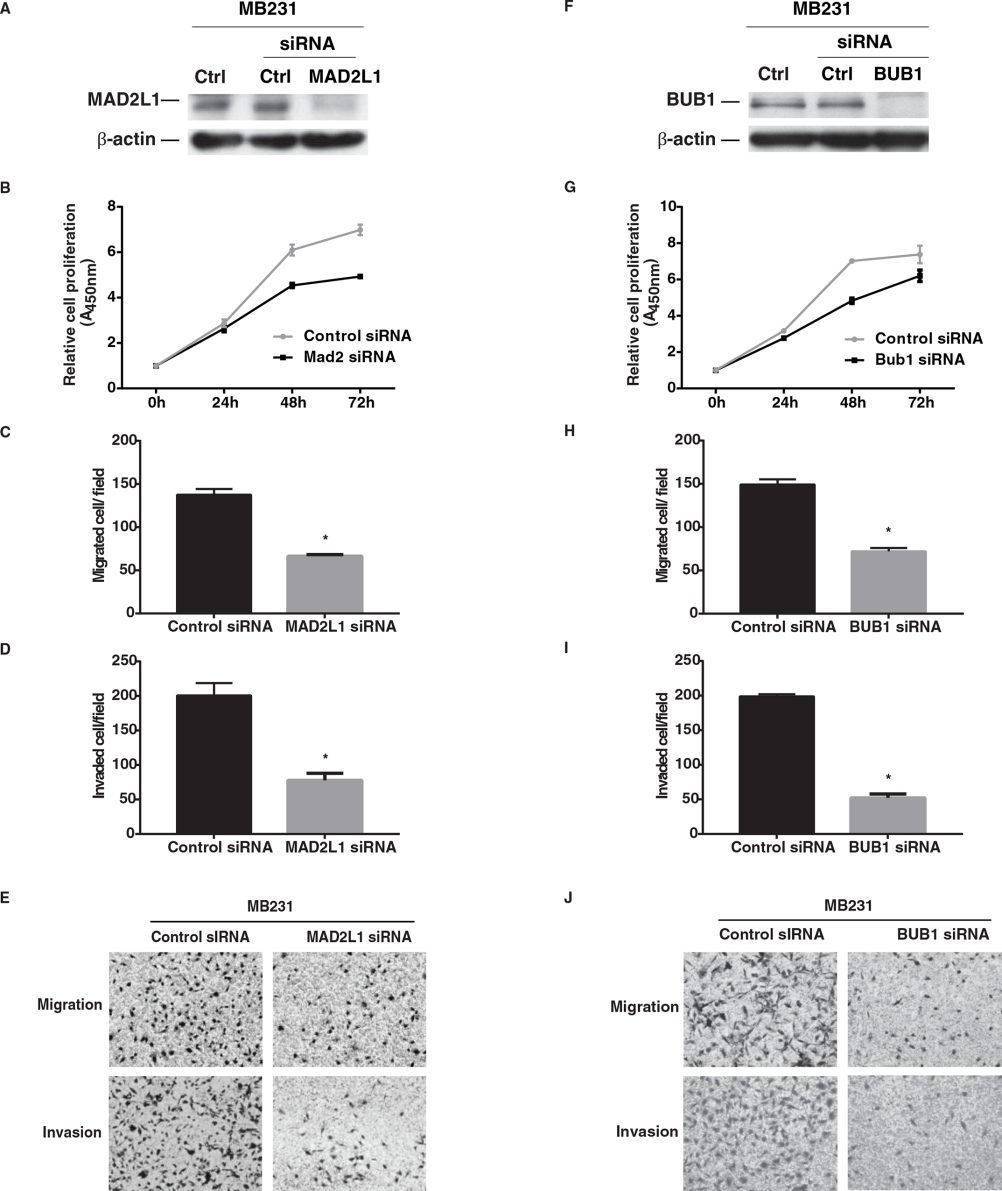
Effects of MAD2L1 or BUB1 depletion on the phenotype of MDA-MB-231 cancer cells *in vitro*. A) MAD2L1 protein expression by western blot analysis, β-actin used as control. B) Knockdown of *MAD2L1* expression inhibited cell proliferation. C) Knockdown of *MAD2L1* expression inhibited cell migration. D) Knockdown of *MAD2L1* expression inhibited cell invasion. E) Representative pictures of cell migration and invasion before and after knockdown. F) BUB1 protein expression by western blot analysis, β-actin used as control. G) Knockdown of *BUB1* expression inhibited cell proliferation. H) Knockdown of *BUB1* expression inhibited cell migration. I) Knockdown of *BUB1* expression inhibited cell invasion. J) Representative pictures of cell migration and invasion before and after knockdown. * = P<0.001.

**Fig 4:**
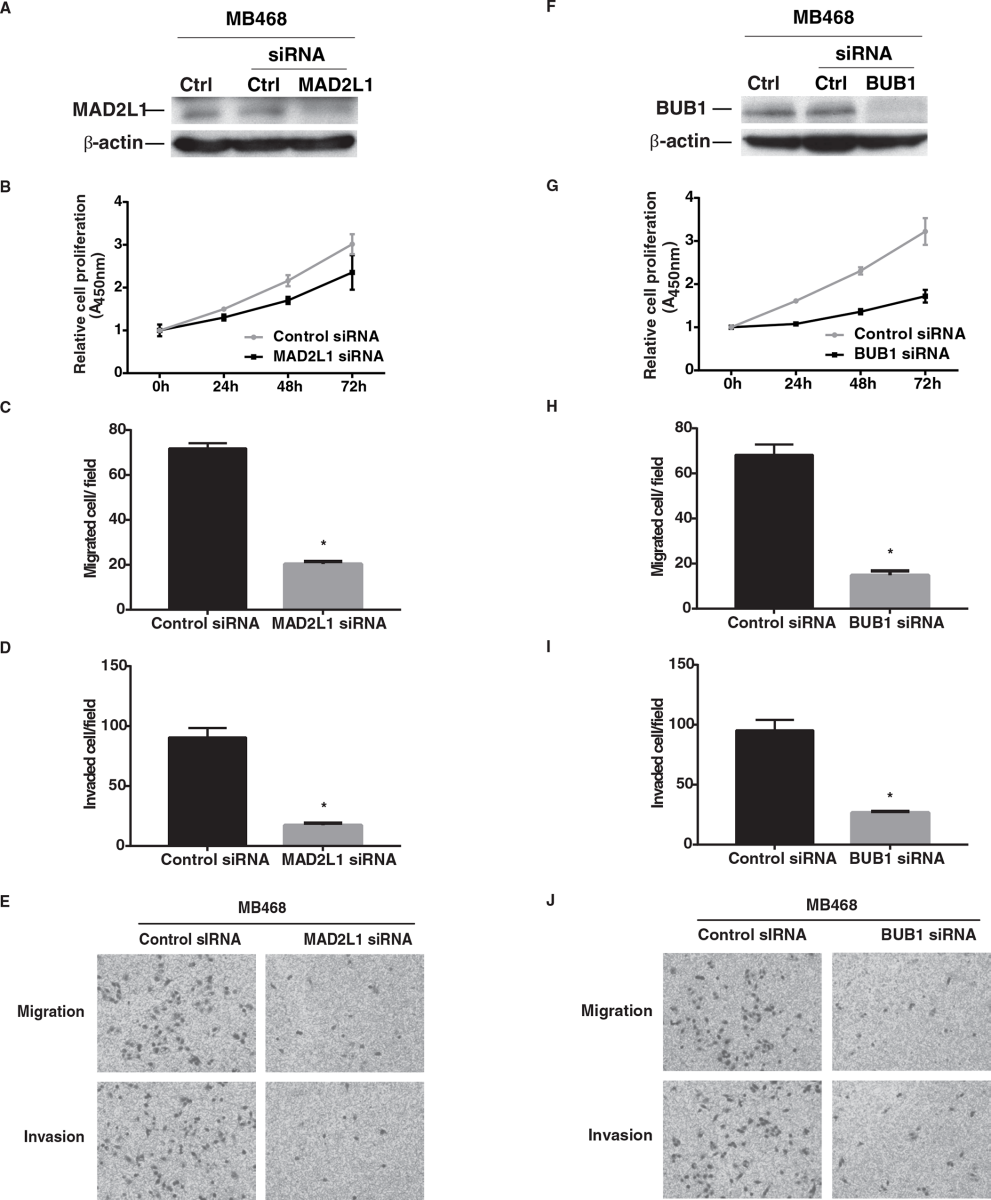
Effects of MAD2L1 or BUB1 depletion on the phenotype of MDA-MB-468 cancer cells *in vitro*. A) MAD2L1 protein expression by western blot analysis, β-actin used as control. B) Knockdown of *MAD2L1* expression inhibited cell proliferation. C) Knockdown of *MAD2L1* expression inhibited cell migration. D) Knockdown of *MAD2L1* expression inhibited cell invasion. E) Representative pictures of cell migration and invasion before and after knockdown. F) BUB1 protein expression by western blot analysis, β-actin used as control. G) Knockdown of *BUB1* expression inhibited cell proliferation. H) Knockdown of *BUB1* expression inhibited cell migration. I) Knockdown of *BUB1* expression inhibited cell invasion. J) Representative pictures of cell migration and invasion before and after knockdown. * = P<0.001.

To evaluate the biological effects of MAD2L1 and BUB1 on breast cancer, we designed two RNA interference (RNAi) assays to knockdown the expression of *MAD2L1* or *BUB1*. The experiments were performed in two triple-negative breast cancer cell lines MDA-MB-231 and MDA-MB-468, which had high expression of endogenous *MAD2L1* and *BUB1*. Cell growth, invasion and migration were evaluated in our *in vitro* experiments. Western blot analyses showed that MAD2L1 and BUB1 proteins were significantly declined after the cells were transfected with siRNAs specific for *MAD2L1* or *BUB1* (Figs 3 and 4). Compared with those transfected with scrambled siRNA, cells with *MAD2L1* or *BUB1* siRNA had lower cell proliferation. Cell invasion and migration were also reduced in those treated with *MAD2L1* or *BUB1* siRNA. Taken together, these results suggest that low *MAD2L1* or *BUB1* expression may inhibit breast cancer cell proliferation, invasion, and migration.

## Discussion

More than a dozen gene expression signatures involving over 1,300 genes have been published for breast cancer prognosis. Among them, no single gene was included in all of the signatures, and only two genes (*MYBL2*, *BIRC5*) were shared by six or seven signatures. These genes were reported to be associated with breast cancer survival, and suppressing the expression of these genes inhibited tumor cell proliferation [31, 32]. Five genes appeared in five signatures, and twelve genes in four signatures. This low frequency of genes overlapping in the signatures suggests that the reported expression signatures are highly heterogeneous. This heterogeneity most likely reflects a diversity of patient populations or tumor specimens with regard to their clinical and pathologic characteristics. It is also possible that some of the results were observed by chance or from false-positive findings. To exclude this possibility, validation of the signature genes in independent studies or distinct patient populations is necessary.

In our study of microarray expression data from 203 breast cancer patients, we analyzed the genes present in multiple signatures, and found three genes (*MAD2L1*, *PTTG1* and *BUB1*) significantly associated with disease-free or overall survival. Of these genes, *PTTG1* has been investigated by Yoon et al. who demonstrated the possible biological relevance of the gene to breast cancer [28]. No reports were found for *MAD2L1* and *BUB1*. Based on the pathway enrichment analysis of the genes involved in multiple expression signatures for prognosis, the cell cycle pathway was suggested to be the most significantly enriched. All the three genes mentioned above are in this pathway.

Genes included in the expression signatures can be involved in multiple biological functions and cellular activities. In the studies exploring gene expression profiles as signatures for survival outcomes, biological and pathologic relevance of the genes to cancer in the signatures are often not evaluated experimentally and validated independently [33]. Breast cancer is a heterogeneous disease characterized by distinct pathologic features, disparate treatment responses, and varied disease outcomes [34]. These differences may reflect specific gene expression signatures. Using the genes in signatures, our pathway enrichment analysis showed that cell cycle is the most significant pathway. As cell cycle involves the processes of cell growth and division, and uncontrolled cell proliferation is a hallmark of cancer, [35, 36], it is not difficult to imagine why these signatures are predictive of disease prognosis. Thus, in this study, we focused on *MAD2L1* and *BUB1*, two critical mitotic checkpoint genes which play an important role in the mitotic process. Our investigation showed that high *MAD2L1* or *BUB1* expression was associated with poor prognosis of breast cancer, and moreover *in vitro* suppression of their expression resulted in reduced cell proliferation and less aggressive cell behavior. In addition, we found from publicly available data that expression of *MAD2L1* and *BUB1* was higher in tumor than in adjacent non-tumor tissues. Collectively, all of these observations suggest that *MAD2L1* and *BUB1*, two genes present in multiple gene expression signatures for breast cancer prognosis, may be important molecules influencing tumor cell activity and patient survival.

Under normal physiologic circumstances, cell cycle is tightly controlled by several mechanisms that regulate cell division and proliferation. One of the primary cell-cycle control mechanisms is the mitotic checkpoints in mitosis [37]. As part of the mitotic checkpoints, the *MAD* (mitotic arrest-deficient) and *BUB* (budding uninhibited by benzimidazole) gene families are essential components of the spindle checkpoint, and were first identified by genetic screening of budding yeast for mutants [38]. *MAD2L1* is required in mitosis when chromosomes are unattached to the mitotic spindle that maintains chromosomal segregation, and is involved in the spindle checkpoint during mitosis [39]. Dysregulation of these genes are associated with chromosomal instability and substantial aneuploidy which occur often in cancer [40, 41]. One study revealed that mRNA levels of many spindle checkpoint genes (*MAD1L1*, *MAD2L1*, *MAD2L2*, *BUB1*, *BUB1B*, *BUB3*, *CDC20* and *TTK*) were almost uniformly increased in breast cancer cell lines relative to the levels in normal breast cells (MCF10A and primary mammary gland); high expression was also observed in high-grade primary breast cancer [42]. In our study, both *MAD2L1* and *BUB1* were expressed substantially higher in high-grade than in low-grade tumors. Our analysis of *MAD2L1* and *BUB1* in two datasets (GSE37751 and GSE29044) also demonstrated higher expression in breast cancer than in adjacent non-tumor tissues.

One review has pointed out that high levels of MAD2L1 or BUB1 could lead to the formation of aggressive tumors in multiple organs [43]. *MAD2L1* has been found to be overexpressed in several types of tumor or cancer cell lines, including breast, lung, liver and stomach [44, 45]. Overexpression of *BUB1* and other family members has been found to be associated with tumor cell proliferation [46]. Transgenic mice overexpressing *BUB1* developed various spontaneous tumors, and showed accelerated myc-induced lymphomagenesis [47]. High expression of *BUB1* was observed in a variety of human malignancies including gastric cancer [46, 48], colorectal cancer [49], and lymphomas [50]. We and others found high expression of *MAD2L1* and *BUB1* in breast cancer and their associations with unfavorable prognosis [2]. Although mounting evidence suggests that high *MAD2L1* or *BUB1* are associated with tumor progression, study findings have been inconsistent. Reduced *BUB1* expression was observed in a subset of pancreatic cancer cells [51]. This inconsistency may arise from different expression in these checkpoint components between actively proliferating cells and quiescent or differentiated cells. Increased *BUB1* expression accords with higher mitotic index in tumors compared to neighboring tissues [47]. Alternatively, variation in function may reflect compensation for other defects in the mitotic checkpoint.

In our in vitro experiments, we also observed that down-regulation of MAD2L1 or BUB1 expression resulted in reduced cell migration and invasion. The underlying mechanism for these effects is unknown. One study showed that MAD2L1 was a direct transcriptional target of E2F, and their interaction could cause retinoblastoma pathway dysregulation [52], which may lead to the suppression of migration and invasion. Another recent study found that knockdown of BUB1 could significantly inhibit the TGF-β-mediated induction of cell migration and invasion [53].

In summary, our study confirmed the prognostic value of two key mitotic checkpoint genes *MAD2L1* and *BUB1*, which have been included in multiple gene expression signatures for breast cancer prognosis. We also found that these genes are biologically relevant to breast cancer progression, as suppression of their expression was associated with reduced tumor cell growth, migration and invasion. Together, our investigation suggests that these genes may serve as tumor markers for breast cancer prognosis as well as potential therapeutic targets to suppress tumor growth.

## Supporting Information

S1 Table Summary of published gene signature included in the current study. (DOCX)

S2 Table List of genes appeared three or more gene signatures. (DOCX)
