# Supplementary material for: Biological and Clinical Significance of MAD2L1 and BUB1, Genes Frequently Appearing in Expression Signatures for Breast Cancer Prognosis
Source: PLoS One. 2015 Aug 19;10(8):e0136246. doi: 10.1371/journal.pone.0136246 (PMC4546117; doi:10.1371/journal.pone.0136246)
Supplement: S1 Table — (DOCX) [file pone.0136246.s001.docx]

S1 Table. Summary of published gene signature included in the current study

| **First author** | **Year** | **Population** | **Platform** | **Gene number** | **References** |
| --- | --- | --- | --- | --- | --- |
| Van 't Veer et al. | 2002 | European | Agilent Human oligo microarrays | 231 | [[1](#_ENREF_1)] |
| Paik et al. | 2004 | European | Oncotype DX assay | 16 | [[2](#_ENREF_2)] |
| Chang et al. | 2004 | European | Stanford 43k cDNA array | 512 | [[3](#_ENREF_3)] |
| Dai .et al.^*^ | 2005 | European | Agilent Human oligo microarrays | 50 | [[4](#_ENREF_4)] |
| Minn et al. | 2005 | European | Affymetrix U133A GeneChip | 54 | [[5](#_ENREF_5)] |
| Pawitan et al. | 2005 | European | Affymetrix U133A GeneChip | 64 | [[6](#_ENREF_6)] |
| Wang et al. | 2005 | European | Affymetrix U133A GeneChip | 76 | [[7](#_ENREF_7)] |
| Chi et al | 2006 | European | Stanford 43k cDNA array | 168 | [[8](#_ENREF_8)] |
| Hu et al. | 2006 | European | Agilent Human oligo microarrays | 306 | [[9](#_ENREF_9)] |
| Sotiriou et al. | 2006 | European | Affymetrix U133A GeneChip | 97 | [[10](#_ENREF_10)] |
| Liu et al. | 2007 | European | Affymetrix U133A GeneChip | 186 | [[11](#_ENREF_11)] |
| Finak et al. | 2008 | European | Agilent Human oligo microarrays | 26 | [[12](#_ENREF_12)] |
| Van Vliet et al.^*^ | 2008 | European | Affymetrix U133A GeneChip | 127 | [[13](#_ENREF_13)] |
| Parker et al. | 2009 | European | Agilent human 1Av2 microarrays or custom-designed Agilent human 22k arrays | 50 | [[14](#_ENREF_14)] |
| Filipits et al. | 2011 | European | RT-qPCR | 63 | [[15](#_ENREF_15)] |
| Yin et al. | 2014 | European | Affymetrix U133A GeneChip | 41 | [[16](#_ENREF_16)] |

* Data from the Van't Veer et al. datase

**Reference**

1. van 't Veer LJ, Dai H, van de Vijver MJ, He YD, Hart AA, Mao M, et al. Gene expression profiling predicts clinical outcome of breast cancer. Nature. 2002;415(6871):530-6. doi: 10.1038/415530a. PubMed PMID: 11823860.

2. Paik S, Shak S, Tang G, Kim C, Baker J, Cronin M, et al. A multigene assay to predict recurrence of tamoxifen-treated, node-negative breast cancer. The New England journal of medicine. 2004;351(27):2817-26. doi: 10.1056/NEJMoa041588. PubMed PMID: 15591335.

3. Chang HY, Sneddon JB, Alizadeh AA, Sood R, West RB, Montgomery K, et al. Gene expression signature of fibroblast serum response predicts human cancer progression: similarities between tumors and wounds. PLoS biology. 2004;2(2):E7. doi: 10.1371/journal.pbio.0020007. PubMed PMID: 14737219; PubMed Central PMCID: PMC314300.

4. Dai H, van't Veer L, Lamb J, He YD, Mao M, Fine BM, et al. A cell proliferation signature is a marker of extremely poor outcome in a subpopulation of breast cancer patients. Cancer research. 2005;65(10):4059-66. doi: 10.1158/0008-5472.CAN-04-3953. PubMed PMID: 15899795.

5. Minn AJ, Gupta GP, Siegel PM, Bos PD, Shu W, Giri DD, et al. Genes that mediate breast cancer metastasis to lung. Nature. 2005;436(7050):518-24. doi: 10.1038/nature03799. PubMed PMID: 16049480; PubMed Central PMCID: PMC1283098.

6. Pawitan Y, Bjohle J, Amler L, Borg AL, Egyhazi S, Hall P, et al. Gene expression profiling spares early breast cancer patients from adjuvant therapy: derived and validated in two population-based cohorts. Breast cancer research : BCR. 2005;7(6):R953-64. doi: 10.1186/bcr1325. PubMed PMID: 16280042; PubMed Central PMCID: PMC1410752.

7. Wang Y, Klijn JG, Zhang Y, Sieuwerts AM, Look MP, Yang F, et al. Gene-expression profiles to predict distant metastasis of lymph-node-negative primary breast cancer. Lancet. 2005;365(9460):671-9. doi: 10.1016/S0140-6736(05)17947-1. PubMed PMID: 15721472.

8. Chi JT, Wang Z, Nuyten DS, Rodriguez EH, Schaner ME, Salim A, et al. Gene expression programs in response to hypoxia: cell type specificity and prognostic significance in human cancers. PLoS medicine. 2006;3(3):e47. doi: 10.1371/journal.pmed.0030047. PubMed PMID: 16417408; PubMed Central PMCID: PMC1334226.

9. Hu Z, Fan C, Oh DS, Marron JS, He X, Qaqish BF, et al. The molecular portraits of breast tumors are conserved across microarray platforms. BMC genomics. 2006;7:96. doi: 10.1186/1471-2164-7-96. PubMed PMID: 16643655; PubMed Central PMCID: PMC1468408.

10. Sotiriou C, Wirapati P, Loi S, Harris A, Fox S, Smeds J, et al. Gene expression profiling in breast cancer: understanding the molecular basis of histologic grade to improve prognosis. Journal of the National Cancer Institute. 2006;98(4):262-72. doi: 10.1093/jnci/djj052. PubMed PMID: 16478745.

11. Liu R, Wang X, Chen GY, Dalerba P, Gurney A, Hoey T, et al. The prognostic role of a gene signature from tumorigenic breast-cancer cells. The New England journal of medicine. 2007;356(3):217-26. doi: 10.1056/NEJMoa063994. PubMed PMID: 17229949.

12. Finak G, Bertos N, Pepin F, Sadekova S, Souleimanova M, Zhao H, et al. Stromal gene expression predicts clinical outcome in breast cancer. Nature medicine. 2008;14(5):518-27. doi: 10.1038/nm1764. PubMed PMID: 18438415.

13. van Vliet MH, Reyal F, Horlings HM, van de Vijver MJ, Reinders MJ, Wessels LF. Pooling breast cancer datasets has a synergetic effect on classification performance and improves signature stability. BMC genomics. 2008;9:375. doi: 10.1186/1471-2164-9-375. PubMed PMID: 18684329; PubMed Central PMCID: PMC2527336.

14. Parker JS, Mullins M, Cheang MC, Leung S, Voduc D, Vickery T, et al. Supervised risk predictor of breast cancer based on intrinsic subtypes. Journal of clinical oncology : official journal of the American Society of Clinical Oncology. 2009;27(8):1160-7. doi: 10.1200/JCO.2008.18.1370. PubMed PMID: 19204204; PubMed Central PMCID: PMC2667820.

15. Filipits M, Rudas M, Jakesz R, Dubsky P, Fitzal F, Singer CF, et al. A new molecular predictor of distant recurrence in ER-positive, HER2-negative breast cancer adds independent information to conventional clinical risk factors. Clinical cancer research : an official journal of the American Association for Cancer Research. 2011;17(18):6012-20. doi: 10.1158/1078-0432.CCR-11-0926. PubMed PMID: 21807638.

16. Yin ZQ, Liu JJ, Xu YC, Yu J, Ding GH, Yang F, et al. A 41-gene signature derived from breast cancer stem cells as a predictor of survival. Journal of experimental & clinical cancer research : CR. 2014;33:49. doi: 10.1186/1756-9966-33-49. PubMed PMID: 24906694.
