# Supplementary material for: Biological and Clinical Significance of MAD2L1 and BUB1, Genes Frequently Appearing in Expression Signatures for Breast Cancer Prognosis
Source: PLoS One. 2015 Aug 19;10(8):e0136246. doi: 10.1371/journal.pone.0136246 (PMC4546117; doi:10.1371/journal.pone.0136246)
Supplement: S2 Table — (DOCX) [file pone.0136246.s002.docx]

S2 Table. List of genes appeared three or more gene signatures

| **Gene name** | **Frequency** | **References** |
| --- | --- | --- |
| *BIRC5* | 7 | [[1-7](#_ENREF_1)] |
| *MYBL2* | 6 | [[1-3](#_ENREF_1),[5](#_ENREF_5),[8](#_ENREF_8),[9](#_ENREF_9)] |
| *BUB1* | 5 | [[2](#_ENREF_2),[5-8](#_ENREF_5)] |
| *CENPF* | 5 | [[2](#_ENREF_2),[3](#_ENREF_3),[5](#_ENREF_5),[7](#_ENREF_7),[10](#_ENREF_10)] |
| *MAD2L1* | 5 | [[2](#_ENREF_2),[5-8](#_ENREF_5)] |
| *PRC1* | 5 | [[2](#_ENREF_2),[6-8](#_ENREF_6),[10](#_ENREF_10)] |
| *RRM2* | 5 | [[2](#_ENREF_2),[3](#_ENREF_3),[7](#_ENREF_7),[8](#_ENREF_8),[10](#_ENREF_10)] |
| *CCNB1* | 4 | [[1-3](#_ENREF_1),[8](#_ENREF_8)] |
| *CCNB2* | 4 | [[2](#_ENREF_2),[6-8](#_ENREF_6)] |
| *CCNE2* | 4 | [[2](#_ENREF_2),[6](#_ENREF_6),[7](#_ENREF_7),[11](#_ENREF_11)] |
| *CENPA* | 4 | [[2](#_ENREF_2),[6-8](#_ENREF_6)] |
| *H2AFZ* | 4 | [[2](#_ENREF_2),[8-10](#_ENREF_8)] |
| *KPNA2* | 4 | [[2](#_ENREF_2),[6](#_ENREF_6),[8](#_ENREF_8),[11](#_ENREF_11)] |
| *MKI67* | 4 | [[2](#_ENREF_2),[3](#_ENREF_3),[8](#_ENREF_8),[10](#_ENREF_10)] |
| *MLF1IP* | 4 | [[2](#_ENREF_2),[8-10](#_ENREF_8)] |
| *PGK1* | 4 | [[7](#_ENREF_7),[8](#_ENREF_8),[12](#_ENREF_12),[13](#_ENREF_13)] |
| *PTTG1* | 4 | [[2](#_ENREF_2),[3](#_ENREF_3),[5](#_ENREF_5),[8](#_ENREF_8)] |
| *STK6* | 4 | [[2](#_ENREF_2),[5-7](#_ENREF_5)] |
| *TRIP13* | 4 | [[2](#_ENREF_2),[6-8](#_ENREF_6)] |
| *ADM* | 3 | [[7](#_ENREF_7),[13](#_ENREF_13),[14](#_ENREF_14)] |
| *ASPM* | 3 | [[2](#_ENREF_2),[8](#_ENREF_8),[10](#_ENREF_10)] |
| *BCL2* | 3 | [[1](#_ENREF_1),[3](#_ENREF_3),[12](#_ENREF_12)] |
| *BM039* | 3 | [[2](#_ENREF_2),[6](#_ENREF_6),[7](#_ENREF_7)] |
| *BUB1B* | 3 | [[2](#_ENREF_2),[8](#_ENREF_8),[10](#_ENREF_10)] |
| *CCT5* | 3 | [[2](#_ENREF_2),[8](#_ENREF_8),[9](#_ENREF_9)] |
| *CDC2* | 3 | [[2](#_ENREF_2),[5](#_ENREF_5),[8](#_ENREF_8)] |
| *CDC20* | 3 | [[2](#_ENREF_2),[3](#_ENREF_3),[8](#_ENREF_8)] |
| *CDKN3* | 3 | [[2](#_ENREF_2),[5](#_ENREF_5),[8](#_ENREF_8)] |
| *CIRBP* | 3 | [[7](#_ENREF_7),[8](#_ENREF_8),[12](#_ENREF_12)] |
| *CKS2* | 3 | [[5](#_ENREF_5),[7](#_ENREF_7),[8](#_ENREF_8)] |
| *CYBRD1* | 3 | [[4](#_ENREF_4),[8](#_ENREF_8),[9](#_ENREF_9)] |
| *DC13* | 3 | [[2](#_ENREF_2),[6](#_ENREF_6),[7](#_ENREF_7)] |
| *DKFZp762E1312* | 3 | [[2](#_ENREF_2),[7](#_ENREF_7),[8](#_ENREF_8)] |
| *EPHX2* | 3 | [[4](#_ENREF_4),[5](#_ENREF_5),[8](#_ENREF_8)] |
| *ERBB2* | 3 | [[3-5](#_ENREF_3)] |
| *ESR1* | 3 | [[3-5](#_ENREF_3)] |
| *FLJ10156* | 3 | [[2](#_ENREF_2),[6](#_ENREF_6),[7](#_ENREF_7)] |
| *FUT8* | 3 | [[5](#_ENREF_5),[7](#_ENREF_7),[9](#_ENREF_9)] |
| *KIAA0101* | 3 | [[5](#_ENREF_5),[8](#_ENREF_8),[10](#_ENREF_10)] |
| *KIF20A* | 3 | [[2](#_ENREF_2),[8](#_ENREF_8),[10](#_ENREF_10)] |
| *KIF2C* | 3 | [[2](#_ENREF_2),[3](#_ENREF_3),[8](#_ENREF_8)] |
| *MAPT* | 3 | [[3](#_ENREF_3),[4](#_ENREF_4),[12](#_ENREF_12)] |
| *MELK* | 3 | [[2](#_ENREF_2),[3](#_ENREF_3),[8](#_ENREF_8)] |
| *MMP1* | 3 | [[4](#_ENREF_4),[5](#_ENREF_5),[15](#_ENREF_15)] |
| *NUSAP1* | 3 | [[2](#_ENREF_2),[8](#_ENREF_8),[10](#_ENREF_10)] |
| *PFKP* | 3 | [[7](#_ENREF_7),[9](#_ENREF_9),[13](#_ENREF_13)] |
| *PTGER3* | 3 | [[4](#_ENREF_4),[5](#_ENREF_5),[8](#_ENREF_8)] |
| *RACGAP1* | 3 | [[2](#_ENREF_2),[4](#_ENREF_4),[8](#_ENREF_8)] |
| *RAI2* | 3 | [[7](#_ENREF_7),[8](#_ENREF_8),[14](#_ENREF_14)] |
| *SOX4* | 3 | [[5](#_ENREF_5),[13](#_ENREF_13),[15](#_ENREF_15)] |
| *SQLE* | 3 | [[4](#_ENREF_4),[8](#_ENREF_8),[9](#_ENREF_9)] |
| *STC2* | 3 | [[4](#_ENREF_4),[12](#_ENREF_12),[13](#_ENREF_13)] |
| *STK15* | 3 | [[1](#_ENREF_1),[6](#_ENREF_6),[7](#_ENREF_7)] |
| *TOP2A* | 3 | [[4](#_ENREF_4),[5](#_ENREF_5),[10](#_ENREF_10)] |
| *UBE2C* | 3 | [[2-4](#_ENREF_2)] |
| *ZWINT* | 3 | [[2](#_ENREF_2),[6](#_ENREF_6),[8](#_ENREF_8)] |

**References**

1. Paik S, Shak S, Tang G, Kim C, Baker J, et al. (2004) A multigene assay to predict recurrence of tamoxifen-treated, node-negative breast cancer. N Engl J Med 351: 2817-2826.

2. Sotiriou C, Wirapati P, Loi S, Harris A, Fox S, et al. (2006) Gene expression profiling in breast cancer: understanding the molecular basis of histologic grade to improve prognosis. J Natl Cancer Inst 98: 262-272.

3. Parker JS, Mullins M, Cheang MC, Leung S, Voduc D, et al. (2009) Supervised risk predictor of breast cancer based on intrinsic subtypes. J Clin Oncol 27: 1160-1167.

4. Filipits M, Rudas M, Jakesz R, Dubsky P, Fitzal F, et al. (2011) A new molecular predictor of distant recurrence in ER-positive, HER2-negative breast cancer adds independent information to conventional clinical risk factors. Clin Cancer Res 17: 6012-6020.

5. Hu Z, Fan C, Oh DS, Marron JS, He X, et al. (2006) The molecular portraits of breast tumors are conserved across microarray platforms. BMC Genomics 7: 96.

6. Dai H, van't Veer L, Lamb J, He YD, Mao M, et al. (2005) A cell proliferation signature is a marker of extremely poor outcome in a subpopulation of breast cancer patients. Cancer Res 65: 4059-4066.

7. van 't Veer LJ, Dai H, van de Vijver MJ, He YD, Hart AA, et al. (2002) Gene expression profiling predicts clinical outcome of breast cancer. Nature 415: 530-536.

8. van Vliet MH, Reyal F, Horlings HM, van de Vijver MJ, Reinders MJ, et al. (2008) Pooling breast cancer datasets has a synergetic effect on classification performance and improves signature stability. BMC Genomics 9: 375.

9. Chang HY, Sneddon JB, Alizadeh AA, Sood R, West RB, et al. (2004) Gene expression signature of fibroblast serum response predicts human cancer progression: similarities between tumors and wounds. PLoS Biol 2: E7.

10. Pawitan Y, Bjohle J, Amler L, Borg AL, Egyhazi S, et al. (2005) Gene expression profiling spares early breast cancer patients from adjuvant therapy: derived and validated in two population-based cohorts. Breast Cancer Res 7: R953-964.

11. Wang Y, Klijn JG, Zhang Y, Sieuwerts AM, Look MP, et al. (2005) Gene-expression profiles to predict distant metastasis of lymph-node-negative primary breast cancer. Lancet 365: 671-679.

12. Liu R, Wang X, Chen GY, Dalerba P, Gurney A, et al. (2007) The prognostic role of a gene signature from tumorigenic breast-cancer cells. N Engl J Med 356: 217-226.

13. Chi JT, Wang Z, Nuyten DS, Rodriguez EH, Schaner ME, et al. (2006) Gene expression programs in response to hypoxia: cell type specificity and prognostic significance in human cancers. PLoS Med 3: e47.

14. Finak G, Bertos N, Pepin F, Sadekova S, Souleimanova M, et al. (2008) Stromal gene expression predicts clinical outcome in breast cancer. Nat Med 14: 518-527.

15. Minn AJ, Gupta GP, Siegel PM, Bos PD, Shu W, et al. (2005) Genes that mediate breast cancer metastasis to lung. Nature 436: 518-524.
